# Sensitive electrochemical sensor based on poly(l-glutamic acid)/graphene oxide composite material for simultaneous detection of heavy metal ions†

**DOI:** 10.1039/c9ra01891c

**Published:** 2019-06-03

**Authors:** Wei Yi, Zihua He, Junjie Fei, Xiaohua He

**Affiliations:** School of Chemistry and Molecular Engineering, East China Normal University 500 Dongchuan Road Shanghai 200241 China xhhe@chem.ecnu.edu.cn; Key Laboratory of Environmentally Friendly Chemistry and Applications of Ministry of Education, College of Chemistry, Xiangtan University Xiangtan 411105 China

## Abstract

Heavy metal pollution can be toxic to humans and wildlife, thus it is of great significance to develop rapid and sensitive methods to detect heavy metal ions. Here, a novel type of electrochemical sensor for the simultaneous detection of heavy metal ions has been prepared by using poly(l-glutamic acid) (PGA) and graphene oxide (GO) composite materials to modify the glassy carbon electrode (GCE). Due to the good binding properties of poly(l-glutamic acid) (PGA) for the heavy metal ions (such as Cu^2+^, Cd^2+^, and Hg^2+^) as well as good electron conductivity of graphene oxide (GO), the heavy metal ions, Cu^2+^, Cd^2+^, and Hg^2+^ in aqueous solution can be accurately detected by using differential pulse anodic stripping voltammetry method (DPASV). Under the optimized experiment conditions, the modified GCE shows excellent electrochemical performance toward Cu^2+^, Cd^2+^, and Hg^2+^, and the linear range of PG/GCE for Cu^2+^, Cd^2+^, and Hg^2+^ is 0.25–5.5 μM, and the limits of detection (LODs, S/N ≥ 3) Cu^2+^, Cd^2+^, and Hg^2+^ are estimated to be 0.024 μM, 0.015 μM and 0.032 μM, respectively. Moreover, the modified GCE is successfully applied to the determination of Cu^2+^, Cd^2+^, and Hg^2+^ in real samples. All obtained results show that the modified electrode not only has the advantages of simple preparation, high sensitivity, and good stability, but also can be applied in the field of heavy metal ion detection.

## Abstract 2

A novel electrochemical sensor with high stability and good reproducibility for the simultaneous detection of heavy metal ions was prepared by using PGA/GO to modify the GCE, showing high sensitivity of superior to most of the reported values.

## Graphical abstract

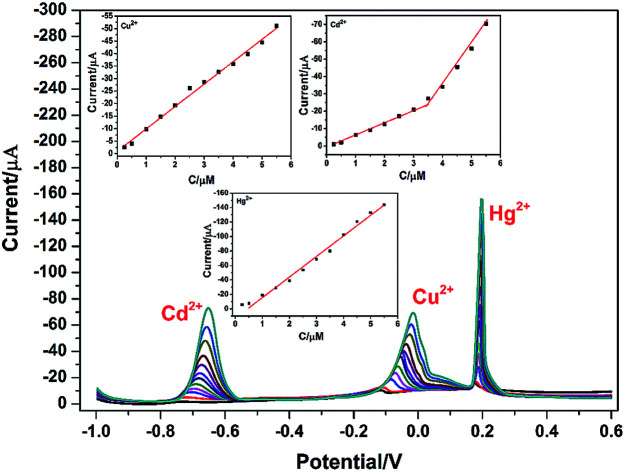

## 1. Introduction

With the development of industry and agriculture, a large amount of heavy metal ions (such as Cu^2+^, Cd^2+^, and Hg^2+^) are discharged into the ecological environment. The heavy metal ions can be accumulated into the human body through drinking water and food chain, severely endangering human health, and have become one of the public great attention focus in recent years.^1^ Hence, developing the effective, inexpensive and rapid heavy metal ion detections are highly urgent. Many methods have been developed and applied into detect the heavy metal ions including atomic absorption spectrometry,^2^ inductively coupled plasma mass spectrometry,^3^ and fluorescence spectrometry,^4^*et al.* However, these classical methods applied in the field of heavy metal ion detection are limited due to their disadvantages of low sensitivity, high cost, and complicated operation. In contrast, the electrochemical methods, especially differential pulse anodic stripping voltammetry (DPASV), have been popularly used to detect the heavy metal ions because of their advantages of high sensitivity, simple operation, rapid analysis and low price.^5^ DPASV is a very sensitive electrochemical analysis method, which is widely applied in the analysis of ultrapure substances and environmental monitoring.^6^ Especially mentioned, composite materials used to modify the working electrodes for the heavy metal ion detections by using DPASV method have been an in-depth study in recent years.^6,7^ For example, Deshmukh and co-workers reported that the determination of the heavy metal ions Cu^2+^, Pb^2+^, and Hg^2+^ on the stainless steel electrode (SS) modified with ethylenediaminetetraacetic acid (EDTA) chelating ligand modified polyaniline^8^ and singe walled carbon nanotubes (SWCNTs) based nanocomposite (EDTA-PANI/SWNCTs), and the limit of detection the modified electrode toward Cu^2+^, Pb^2+^, and Hg^2+^ was determined as 0.08 μM, 1.65 μM and 0.68 μM, respectively.^9^ EDTA chelating ligand grafted onto PANI and SWCNTs improved the binding ability of heavy metal ions and the conductivity of the materials, respectively. At the same time, the research results also showed that the electron-conducting ability and the binding ability toward heavy metal ions of the composite materials are the key factors affecting the sensitivity of the modified electrode.

As a representative of 2D carbon materials, graphene oxide (GO) has been widely used to detect heavy metal ions in electrochemical analysis region.^7*b*,8,10^ This is mainly due to its good hydrophobicity, moderate conductivity, high chemical stability and excellent electrochemical properties, which is beneficial to improve the detection effect. Lu *et al.* prepared a series of heavy metal ions electrochemical sensors using reduced graphene oxide (rGO) material.^11^ Recently, our research group also reported the preparation of the reversible switched pH-responsive hydroquinone electrochemical sensor based on composite film of polystyrene-*b*-poly(acrylic acid)/GO.^12^ On the other hand, poly(amino acid)s and their derivatives or analogues have also been applied for the development of bio/chemical sensors.^5*f*,13^ This is because poly (amino acid)s contain a large number of functional groups (such as carboxyl, amino), which can efficiently bind the detected substances (such as metal ions), thus improving the efficiency of their detection. Lin and co-workers reported a novel biosensor harnessing a peptide layer which has specific affinity to lead ion proved to be highly effective for electrochemical analysis of lead ions.^13*b*^ Benvidi *etc.* reported a novel sensitive electrochemical aptasensor based on poly (l-glutamic acid)/MWCNTs modified glassy carbon electrode for determination of tetracycline, which the combination of poly (l-glutamic acid)/MWCNTs is benefit to improve the detection limit of tetracycline.^13*c*^ However, according to our best knowledge, the detection of heavy metal ions with poly (amino acid)s/GO modified the electrode has been not reported.

**Scheme 1:**
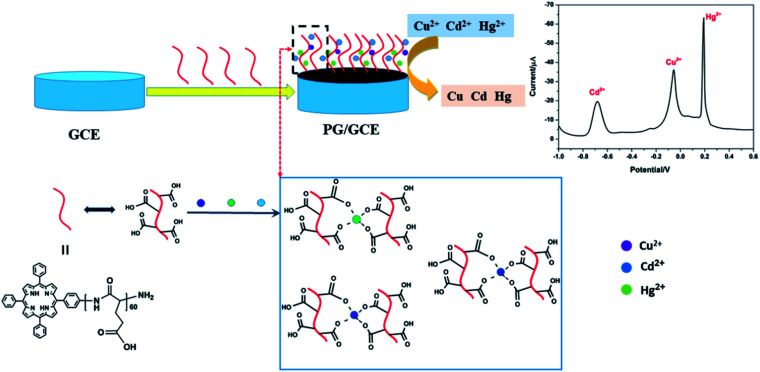
Illustration the mechanism of Cu^2+^, Cd^2+^, and Hg^2+^ detection.

Herein, we prepared a novel type of electrochemical sensor for the simultaneous detection of heavy metal ions Cu^2+^, Cd^2+^, and Hg^2+^, which was constructed through using poly(l-glutamic acid) (PGA) and graphene oxide (GO) composite materials to modify the glassy carbon electrode (GCE). Interest of the constructed electrochemical sensor originated from the following criteria: the ability to bind heavy metal ions with poly(l-glutamic acid) (PGA) due to many carboxyl groups on the side chain of PGA, the conductivity and chemical stability of GO, and their easy preparation and low-cost. In order to better improve the interaction between PGA with GO, porphyrin was introduced into PGA as a terminal group (see ESI, Scheme 1†), which porphyrin with a conjugate ring structure can interact statically with the conductive materials (graphene, carbon nanotubes) by π–π stacking interaction.^14^ The modified GCE with PGA/GO composite materials can used to quantitatively detect heavy metal ions Cu^2+^, Cd^2+^, and Hg^2+^ with both high sensitivity and wide linear detection range. Moreover, the modified electrode can be also used for the content analysis of Cu^2+^, Cd^2+^, and Hg^2+^ in practical samples, which can be a promising electrode for practical applications in heavy-metal-ion detection.

## 2. Experimental section

### 2.1. Reagents and materials

Graphene oxides (GO) was prepared in accordance with the method reported in the literature.^15^ Poly (γ-benzyl-glutamate) with a terminal porphyrin group was prepared by using 5-(4-aminophenyl)-10,15,20-tris(phenyl)porphyrin as an initiator according to the literature,^16^ poly (l-glutamic acid) (PGA) was synthesized according to a published procedure,^17^ and their detailed synthesis process were provided in the ESI.† CuSO_4_, CdSO_4_, trifluoroacetic acid (TFA) were purchased from Aladdin Co. and used without further purification. HgCl_2_ was purchased from Sinopharm Chemical Reagent CO. Ltd (China) and used without further purification. *N*,*N*-Dimethylformamide (DMF) and other reagents used were of analytical grade and purchased from Shanghai Chemical Reagent Company of China. Ultrapure water (18.2 Ω) was used in the process of experiment.

### 2.2. Fabrication of the modified electrodes

Glassy carbon electrodes (GCEs) (*Φ* = 3 mm) were carefully polished with 0.3 μm alumina and 0.05 μm alumina in turn, and then treated in ethanol and ultrapure water for 3 minutes in an ultrasonic bath. Finally, GCEs were dried in the air before use.

PGA/GO (PG) suspension solution was prepared by mixing 1.0 mL of PGA solution (10.0 mg mL^−1^) in DMF and 1.0 mL of GO solution (3.0 mg mL^−1^) in DMF, and sonicating the mixture for 5 minutes. The modified working electrode, PG/GCE, was prepared by dropping 6 μL PG suspension solution on a clean GCE surface and dried in vacuum at room temperature. For comparison, other modified electrodes, GO/GCE and PGA/GCE, were also prepared by the similar method by dropping GO solution and PGA solution, respectively.

### 2.3. Characterizations

Electrochemical experiments were carried out on a CHI 630 Electrochemical Workstation (Shanghai Chenhua Co., Ltd.) with a three-electrode system, consisting of a reference electrode of Ag/AgCl electrode, a platinum wire auxiliary electrode, and a corresponding modified GCE. The electrochemical impedance spectroscopy (EIS) measurements were performed in a glass cell filled with 5 × 10^−3^ M Fe(CN)_6_^3−^/Fe(CN)_6_^4−^ (1 : 1) + 0.1 M KCl solution. Phosphate buffer solutions (PBS) were used as the supporting electrolyte. The whole electrochemical measurements were carried on air atmosphere. Hitachi S-4800 field-emission scanning electron microscopy with an accelerating voltage of 10.0 kV was used to observe the surface morphologies of different films and samples were coated with a gold layer. ^1^H NMR (500 MHz) spectrum was recorded on the Bruker DPX500 spectrometer using TMS as the internal standard of CDCl_3_.

## 3. Results and discussion

### 3.1. SEM characterization

**Fig. 1:**
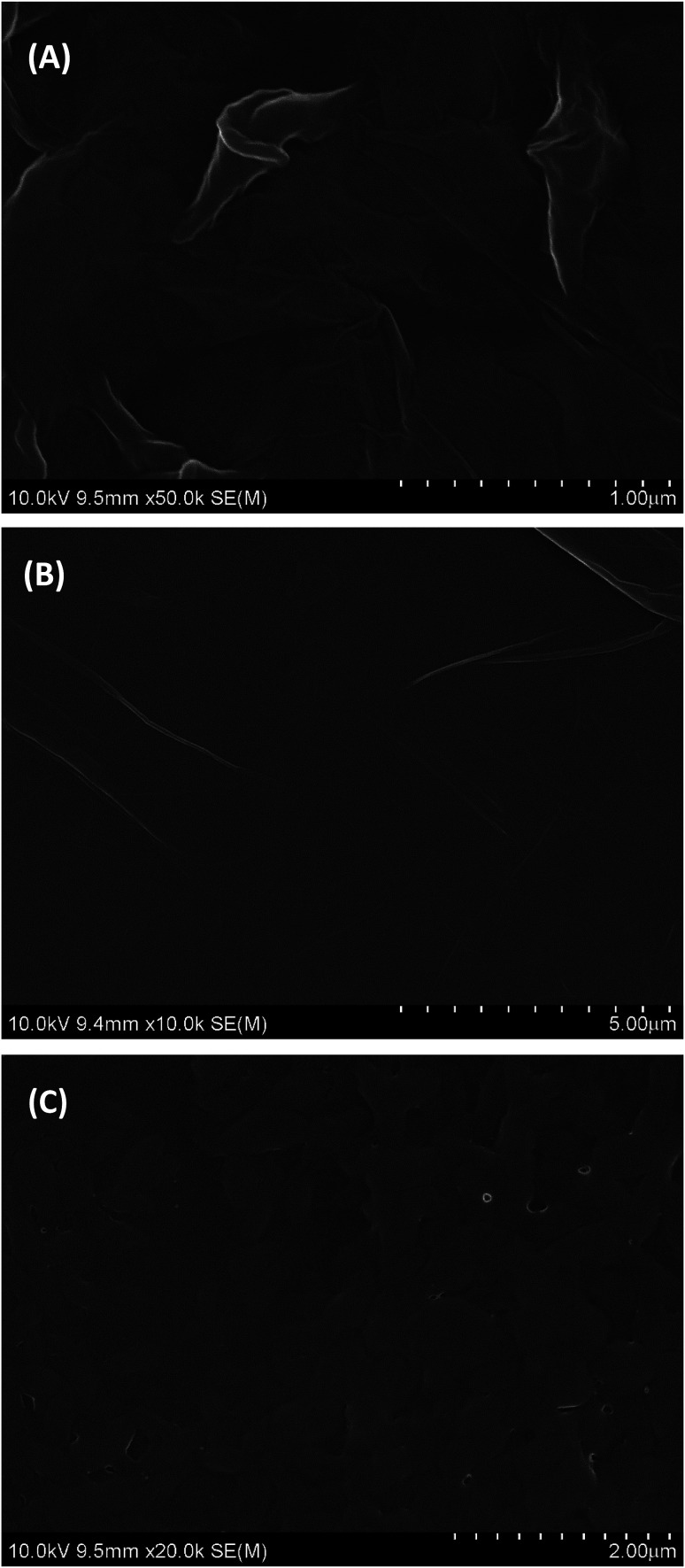
SEM of (A) GO, (B) PGA film, and (C) PG composite film.

The surface morphology of GO, PGA and PG films was investigated by SEM and the SEM images are depicted in Fig. 1. As shown in Fig. 1A, GO shows a smooth surface morphology with many fold structures. PGA is a homogeneous composite film with some small pleated structures (Fig. 1B), indicating its good film-forming property. On the surface of PG films, approximate uniform surfaces without macroscopic phase separation can be observed (Fig. 1C), indicating that they are well composited. This may be due to the strong interactions between some polar groups (such as hydroxyl, carboxyl groups, *etc.*) in GO and PGA chains.

### 3.2. EIS of different electrodes

**Fig. 2:**
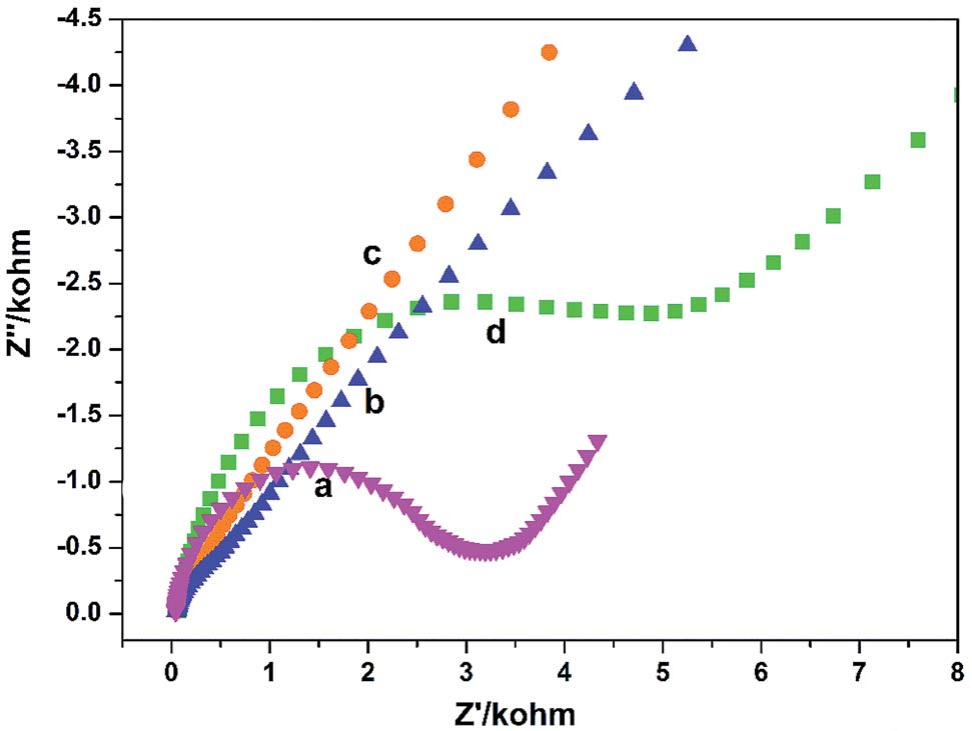
EIS of (a) bare GCE, (b) PG/GCE, (c) GO/GCE, and (d) PGA/GCE in supporting electrolyte. Supporting electrolyte: 10 mmol L^−1^ K_3_[Fe(CN)_6_]/K_4_[Fe(CN)_6_] (1 : 1) + 0.1 mol L^−1^ KCl solution.

The electron transfer properties of the electrodes were analyzed by electrochemical impedance spectroscopy (EIS). The semicircle diameter in the Nyquist plots directly reflects the electron transfer resistance (*R*_et_) at the electrode surface.^18^Fig. 2 shows the results of EIS of different electrodes (Nyquist plots) toward soluble redox probes [Fe(CN)_6_]^3−/4−^. Compared with the bare GCE (Fig. 2a), the redox process of the probes on the GO/GCE (Fig. 2c) hardly displayed electron transfer resistance, indicating that the prepared GO processes good electrical conductivity and the GO/GCE modified by GO has a faster electron transfer rate. At the same time, it also shows that GO can be successfully coated on the surface of the GCE. After modified the GCE by PGA, The semicircular diameter of the PGA/GCE (Fig. 2d) is much larger than that of the GCE (Fig. 2a), meaning that the *R*_et_ value of the PGA/GCE is much larger than that of the GCE, which is due to the poor conductivity of PGA. The semicircular diameter of the PG/GCE (Fig. 2b) is much smaller than that of the PGA/GCE, suggesting that the electron transfer resistance of the redox probe on the PG/GCE surface is much smaller than that of the PGA/GCE, which should be attributed to the good conductivity of GO. These results demonstrate that the PG/GCE electrode has been successfully prepared.

**Fig. 3:**
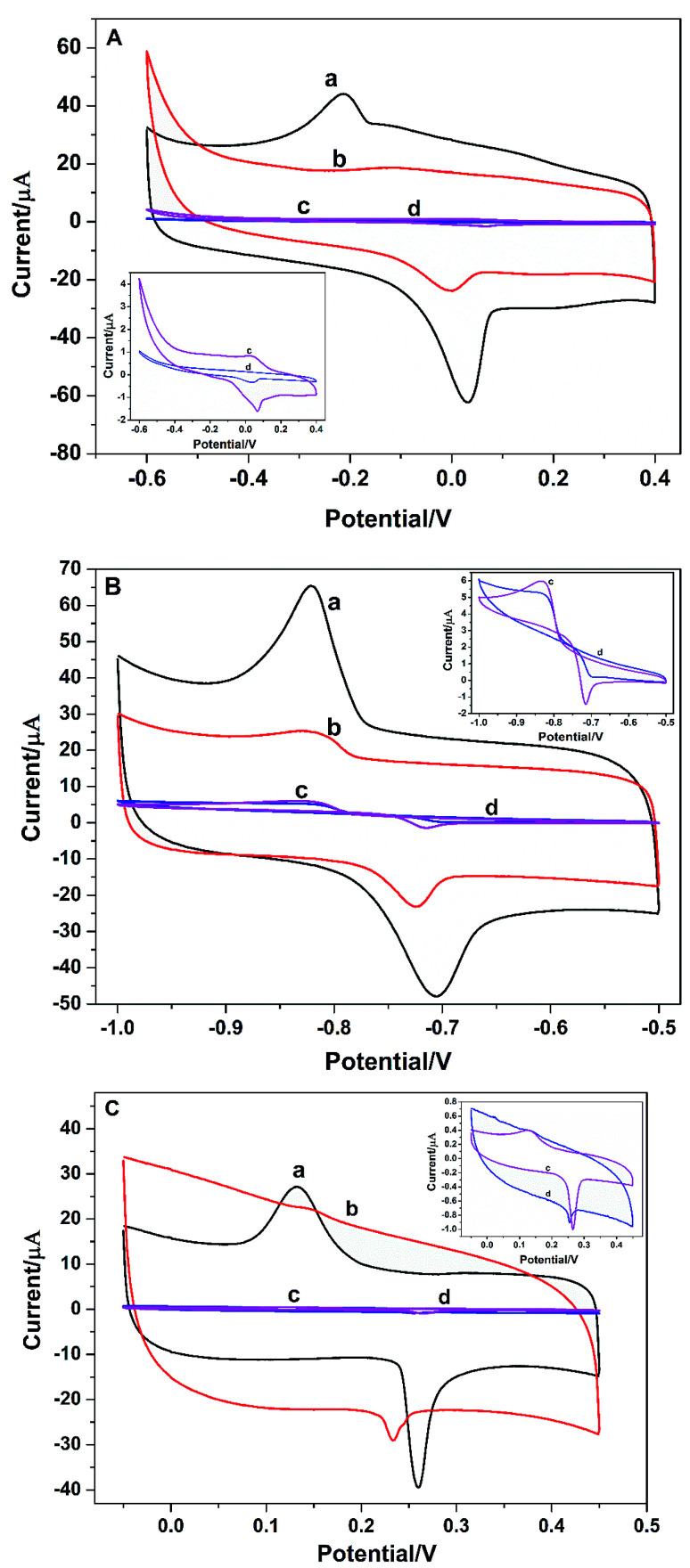
(A) Cyclic voltammograms of 3.5 μM Cu^2+^ at 0.1 V s^−1^ for (a) PG/GCE, (b) GO/GCE, (c) bare GCE, (d) PGA/GCE in 0.1 M PBS buffer (pH 6.5). (B) Cyclic voltammograms of 3.5 μM Cd^2+^ at 0.1 V s^−1^ for (a) PG/GCE, (b) GO/GCE, (c) bare GCE, (d) PGA/GCE in 0.1 M PBS buffer (pH 6.5). (C) Cyclic voltammograms of 2.0 μM Hg^2+^ at 0.1 V s^−1^ for (a) PG/GCE, (b) GO/GCE, (c) bare GCE, (d) PGA/GCE in 0.1 M PBS buffer (pH 6.5).

Electrochemical behaviors of metal ions at the bare GCE, PGA/GCE, GO/GCE, and PG/GCE were investigated by the cyclic voltammetry (CV). Fig. 3 depicts CV responses of Cu^2+^, Cd^2+^ and Hg^2+^ at the four electrodes in 0.1 mol L^−1^ PBS buffer (pH 6.5) at a scan rate of 100 mV s^−1^. Compared with PG/GCE and GO/GCE (Fig. 3 curve a and curve b), CV responses are severely suppressed at the bare GCE and PGA/GCE (Fig. 3 curve c and curve d), which is mainly attributed the good electronic conductivity of GO, thus enhancing the electro-catalytic ability of the modified electrodes for the three metal ions. On the other hand, a pair of redox peaks on bare GCE (Fig. 3 curve c) are bigger than that on PGA/GCE (Fig. 3 curve d), indicating the poor conductivity of PGA. The results are also consistent with the EIS results. The anodic and cathodic peak currents at PG/GCE (Fig. 3 curve a) can be obviously observed and are much larger than those at GO/GCE (Fig. 3 curve b), demonstrating that the PG composite film enhances the electrochemical response and promotes the electronic transfer property of the PG/GCE electrode, which is mainly attributed the good binding ability between heavy metal ions with PGA due to many carboxyl groups on the side chain of PGA and the good interaction between the porphyrin terminal groups of PGA with GO. Moreover, the obviously increased current signals also proves that the PG film on the surface of the modified electrode, PG/GCE, is suitable for heavy metal ion detection, which is beneficial to increase the regional concentration and the detection limit of trace heavy metal ion. From the research results, PG/GCE has better electro-catalytic performance toward Cu^2+^, Cd^2+^, and Hg^2+^. Therefore, PG/GCE exhibits the applicability towards Cu^2+^, Cd^2+^, and Hg^2+^ detection.

**Fig. 4:**
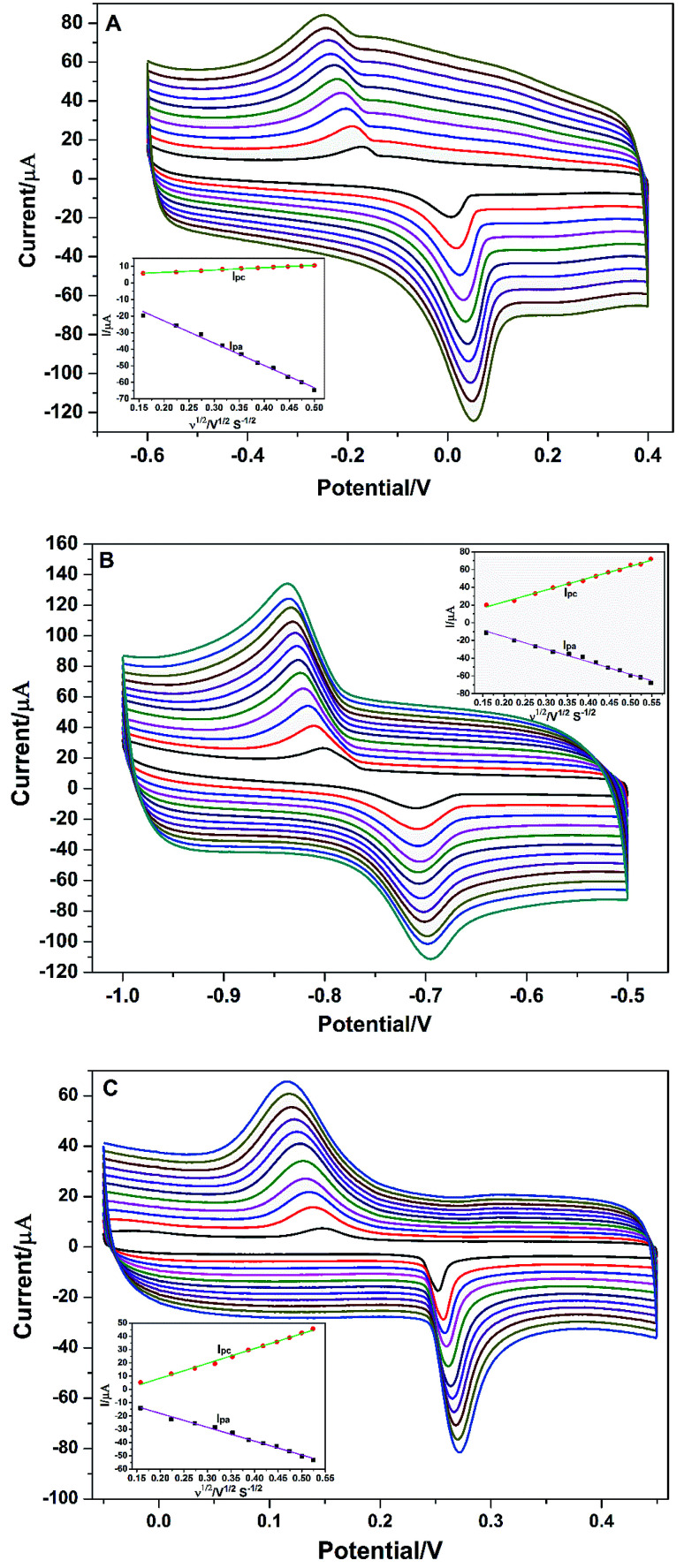
(A) Cyclic voltammograms of 4.0 μM Cu^2+^ in 0.1 M PBS (pH 6.5) at different scan rates on PG/GCE. (B) Cyclic voltammograms of 4.0 μM Cd^2+^ in 0.1 M PBS (pH 6.5) at different scan rates on PG/GCE. (C) Cyclic voltammograms of 2.0 μM Hg^2+^ in 0.1 M PBS (pH 6.5) at different scan rates on PG/GCE. Inset: plot of peak currents (*I*_pa_ and *I*_pc_) against the scan rate (*ν*).

To better understand the redox reaction of Cu^2+^, Cd^2+^, and Hg^2+^ at PG/GCE, the effect of different scan rates on their redox peak currents was also performed by CV and the research results are shown in Fig. 4A series of well-define quasi-reversible redox waves are obviously observed (Fig. 4), and both the redox peak currents and the peak (*E*_pa_: an anodic peak potential)-to-peak (*E*_pc_: a cathodic peak potential) separations (Δ*E*_p_: Δ*E*_p_ = *E*_pa_ − *E*_pc_) increase with increasing the scan rates. As seen from Fig. 4A, the anodic peak current (*I*_pa_) and cathodic peak current (*I*_pc_) at PG/GCE in the potential range of −0.6 V and 0.4 V for Cu^2+^ increase linearly proportional to the square root of the scan rates ranging from 0.025 to 0.25 V s^−1^ (inset of Fig. 4A), and linear regression equations between the electrode peak currents and the square root of the corresponding scan rates (*ν*^1/2^) are expressed as *I*_pa_ = −4.03 − 134.58*ν*^1/2^ (*R*^2^ = 0.993) and *I*_pc_ = −3.60 + 14.20*ν*^1/2^ (*R*^2^ = 0.995),where *R* is the correlation coefficient. The results indicate that the electrochemical process of Cu^2+^ at PG/GCE is an adsorption control process.^19^Fig. 4B shows the relationship between the redox currents of Cd^2+^ and the scan rates at PG/GCE in the potential range of −1.0 V and −0.5 V. With the increase of the scan rates, the electrode peak potentials of Cd^2+^ redox peaks hardly shift, but the electrode peak currents gradually increase. Electrode peak currents (*I*_pa_ and *I*_pc_) have a good linear relationship with the square root of the scan rates (*ν*^1/2^) at the scan rates ranging from 0.025 to 0.30 V s^−1^ (inset of Fig. 4B), and the linear equation between the electrode peak currents and the corresponding *ν*^1/2^ are expressed as *I*_pa_ = 12.86 − 142.01*ν*^1/2^ (*R*^2^ = 0.988) and *I*_pc_ = −3.37 + 134.30*ν*^1/2^ (*R*^2^ = 0.994). The results reveal that the electrochemical process of Cd^2+^ at PG/GCE is also controlled by an adsorption process. The electrochemical behavior of Hg^2+^ at PG/GCE was also performed by CV and shown in Fig. 4C. Very similar results can be obtained (Fig. 4C) and the electrode peak currents (*I*_pa_ and *I*_pc_) at PG/GCE in the potential range of −0.05 V and 0.45 V for Hg^2+^ increase linearly proportional to the square root of the scan rates (*ν*^1/2^) ranging from 0.025 to 0.275 V s^−1^ (inset of Fig. 4C), which are expressed *I*_pa_ = 2.75 − 104.30*ν*^1/2^ (*R*^2^ = 0.995) and *I*_pc_ = −13.86 + 111.76*ν*^1/2^ (*R*^2^ = 0.993), indicating that an adsorption-controlled process occur during the electrochemical process of Hg^2+^ at PG/GCE.

### 3.3. Optimization of experimental parameters

It was well known that the differential pulse anodic stripping voltammetry (DPASV) of the target heavy metal ions were sensitively dependent on the experimental analytical parameters, such as deposition potential, deposition time and pH of the supporting electrolyte. In order to obtain the best voltammetric responses of the PG/GCE towards the simultaneous detection of Cu^2+^, Cd^2+^, and Hg^2+^ by DPASV, three stripping analytical parameters such as deposition potential, deposition time and pH of the supporting electrolyte were fully investigated.

**Fig. 5:**
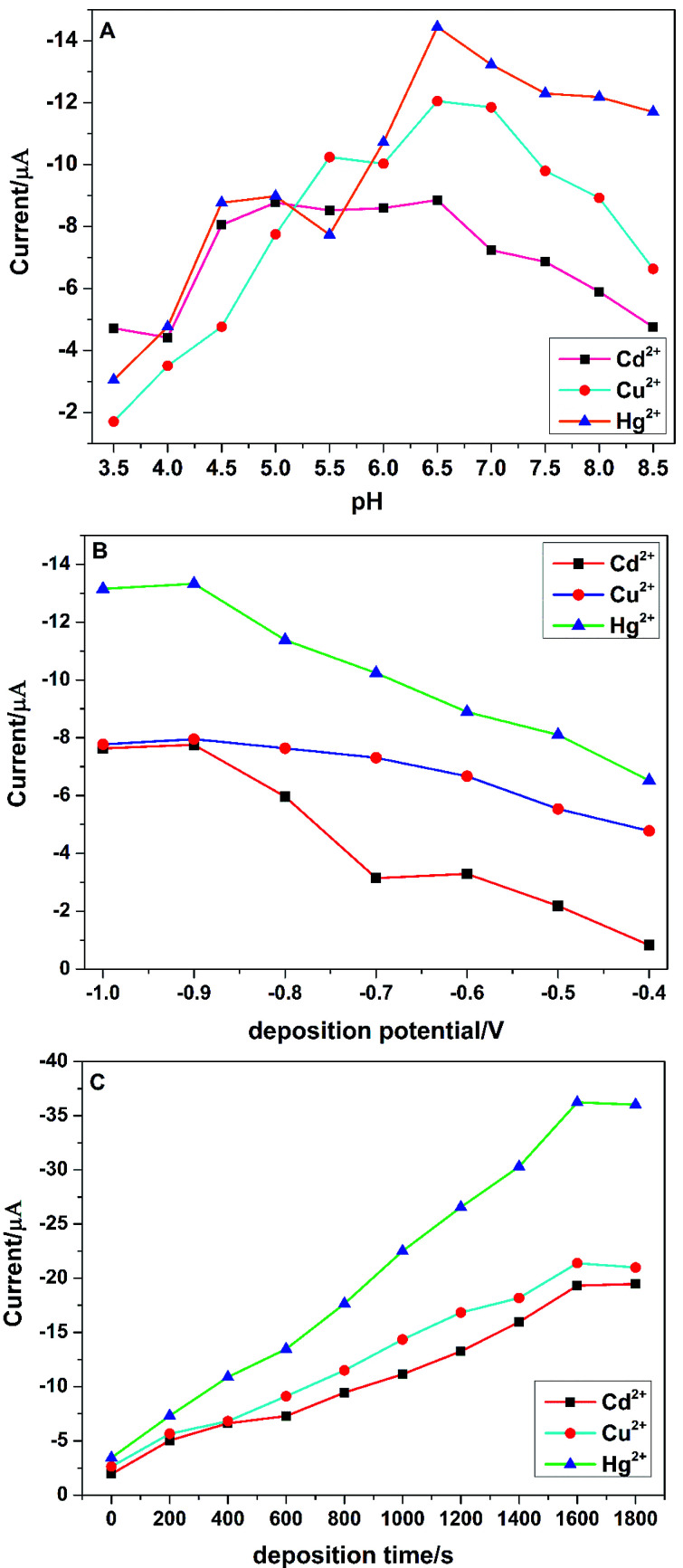
(A) Effect of the electrolyte pH on the peak current of 2.0 μM Cu^2+^, Cd^2+^ and Hg^2+^ in 0.1 M PBS (deposition potential: −0.9 V, deposition time: 400 s). (B) Effect of the deposition potential on the peak current of 2.0 μM Cu^2+^, Cd^2+^ and Hg^2+^ in 0.1 M PBS (pH 6.5) (deposition time: 400 s). (C) Effect of the deposition time on the peak current of 2.0 μM Cu^2+^, Cd^2+^ and Hg^2+^ in 0.1 M PBS (pH 6.5) (deposition potential: −0.9 V).

pH of the supporting electrolyte is another critical factor for the electrochemical detection of heavy metal ions. The effect of the supporting electrolyte pH on the tripping peak currents of Cu^2+^, Cd^2+^, and Hg^2+^ was examined from 3.5 to 8.5 and shown in Fig. 5A. The peak currents of the three target heavy metal ions increased with the increase of pH from 3.5 to 6.5, which may be due to the protonation of carboxylic groups at lower pH values and the decrease of their binding ability to metal ions.^5*f*^ However, the peak currents began to decrease when pH increased from 6.5 to 8.5, which could be attributed to the formation of metal ions hydroxide complexes and the inhibition of their accumulation.^20^ Therefore, the supporting electrolyte at pH 6.5 was chosen throughout the experiments.

The effect of the deposition potential on the stripping responses of Cu^2+^, Cd^2+^, and Hg^2+^ in 0.1 M PBS over the potential range of −1.0 V to −0.4 V after 200 s accumulation was investigated and shown in Fig. 5B. The biggest stripping currents are obtained at −0.9 V for the three heavy metal ions and an obvious decrease of the stripping currents with a more positive deposition potential is observed, which may be attributed to the incomplete reduction of metal ions at a more positive potential.^6^ Therefore, −0.9 V was chosen for the following experiments with high sensitivity and reproducibility. The effect of deposition time on the tripping peak currents was also explored from 0 s to 1800 s and shown in Fig. 5C. The stripping peak currents increased almost proportionally with the increase of the deposition time from 0 s to 1600 s, indicating that a longer deposition time would be benefit for the accumulation of metal ions on the surface the PG/GCE. However, when the deposition time was more than 1600 s, the peak currents level off or slightly decreased, which may be due to the saturation of the surface active sites.^20^ Thus, the deposition time was selected as 1600 s for the simultaneous detection of Cu^2+^, Cd^2+^, and Hg^2+^.

### 3.4. Analytical performance for simultaneous detection of Cu^2+^, Cd^2+^, and Hg^2+^

**Fig. 6:**
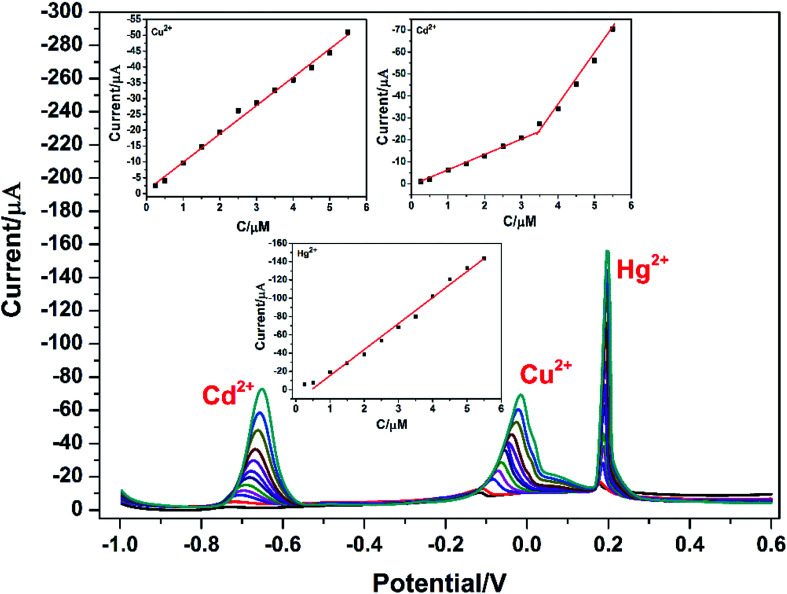
DPASV for different concentrations of Cu^2+^, Cd^2+^, and Hg^2+^ at PG/GCE. The inset is a plot of the peak oxidation current value against its concentration.

Under the above optimized experimental parameters, DPASV measurements were carried out at PG/GCE for simultaneous detection of Cu^2+^, Cd^2+^ and Hg^2+^ by changing their concentrations from 0.25 to 5.50 μM, but keeping at a same concentration in their mixed solution for per detection. As shown in Fig. 6, three well-defined peaks without any overlapping for Cd^2+^, Cu^2+^ and Hg^2+^ are obviously observed at −0.65 V, −0.015 V and 0.2 V, respectively. The peak currents increase linearly *versus* the Cu^2+^ and Hg^2+^ concentrations from 0.25 to 5.50 μM (insets of Fig. 6) and the correction equations are *y* = −0.939 − 8.95*x* (*R*^2^ = 0.993) and *y* = 13.023 − 28.454*x* (*R*^2^ = 0.995) (*y*: current/μA, *x*: concentration/μM), respectively. The limit of detections (LOD) (S/N = 3) are calculated to be 0.024 μM for Cu^2+^ and 0.032 μM for Hg^2+^. However, as seen from the inset of Fig. 6, the analytic curves for Cd^2+^ covered two linear ranges varying from 0.25 to 3.5 μM and 3.5 to 5.5 μM, and the corresponding correction equations are *y* = 1.329 − 7.291*x* (*R*^2^ = 0.995) and *y* = 50.76 − 21.65*x* (*R*^2^ = 0.982), respectively.^6^ The limit of detection (LOD) for Cd^2+^ are calculated to be 0.015 μM and 0.009 μM, respectively. It may be attributed to the two linear regions observed for Cd^2+^ that a dynamic equilibrium for Cd^2+^ adsorption and/or deposition can be gradually reached on the surface of PG/GCE.^21^Table 1 summarized the comparison of the analytical performance for simultaneous detection of Cu^2+^, Cd^2+^ and Hg^2+^ at PG/GCE and that at other electrochemical sensors reported in recent years. In comparison with the reported values, PG/GCE shows both a wide linear range and a lower detection limit for the detection of Cu^2+^, Cd^2+^ and Hg^2+^. The excellent analysis performance of PG/GCE may be attributed to the following factors: (i) PGA has a strong affinity for Cu^2+^, Cd^2+^, and Hg^2+^ due to many carboxyl groups, which is beneficial for the preconcentration of Cu^2+^, Cd^2+^, and Hg^2+^ to the surface of the modified electrode, as shown in Scheme 1. (ii) GO in the composite film has good conductivity, so it can quickly transfer electrochemical signals. (iii) The introduction of porphyrin as a terminal group into PGA better enhances the interaction between PGA and GO, and further improves the electron transfer.

**Table 1:** Comparison the analytical performances of PG/GCE electrode with other reported electrodes for the determination of Cu^2+^, Cd^2+^, and Hg^2+^^a^

Modified electrode	Analytes	Linear range (μM)	LOD (μM)	Reference
EDTA-PANI/SWCNTs/SS	Cu^2+^	1.2–2000	0.08	9
	Hg^2+^	2–2000	0.68	
NH_2_-MIL-88(Fe)-rGO/GCE	Cu^2+^	0.005–0.05	0.0036	7*c*
	Cd^2+^	0.005–0.3	0.0049	
RGO/Bi/CPE	Cu^2+^	0.3125–1.5625	0.406	8
	Cd^2+^	0.1785–1.0714	0.025	
Alk-Ti_3_C_2_/GCE	Cu^2+^	0.1–1.5	0.032	20
	Cd^2+^	0.1–1.5	0.098	
	Hg^2+^	0.1–1.5	0.130	
Pd/PAC/GCE	Cu^2+^	0.5–5.0	0.066	22
	Cd^2+^	0.5–5.5	0.041	
	Hg^2+^	0.24–7.5,	0.054	
NG/GCE	Cu^2+^	0.01–5	0.005	7*d*
	Cd^2+^	0.05–1000	0.05	
	Hg^2+^	0.2–9	0.05	
PG/GCE	Cu^2+^	0.25–5.5	0.024	This work
	Cd^2+^	0.25–5.5	0.015	
	Hg^2+^	0.25–5.5	0.032	

a EDTA: ethylenediaminetetraacetic acid. SS: stainless steel. Alk-Ti_3_C_2_: alkaline-Ti_3_C_2_. PAC: porous activated carbons. NG: N-doped graphene.

### 3.5. Interference performance of PG/GCE

**Fig. 7:**
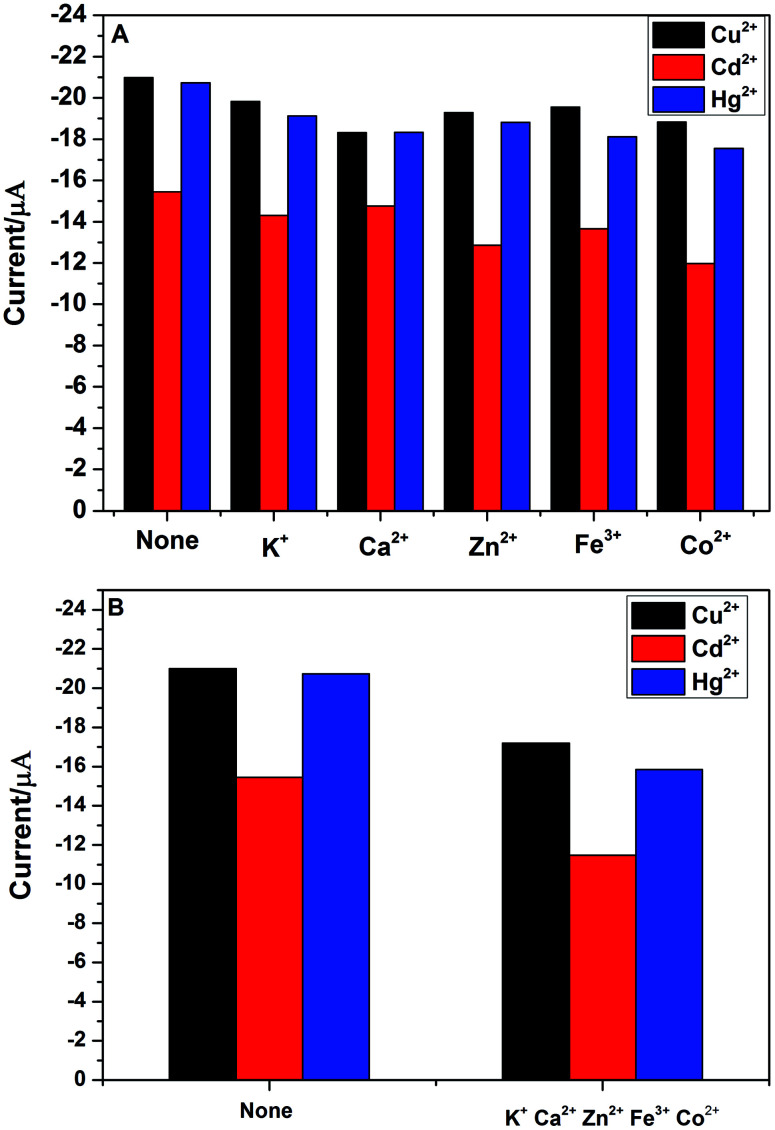
(A) Effect of each interfering metal ions on detection of 2.0 μM Cu^2+^, Cd^2+^ and Hg^2+^ in 0.1 M PBS (pH 6.5). (B) Effect of five interfering metal ions on detection of 2.0 μM Cu^2+^, Cd^2+^ and Hg^2+^ in 0.1 M PBS (pH 6.5).

In practical detection, the interference ions might be co-deposit with the target metal ions. Thus, the electrode selectivity is very important for the analytical detection of heavy metal ions. In order to explore the selectivity of PG/GCE for simultaneous detection of Cu^2+^, Cd^2+^ and Hg^2+^, five usual kinds of foreign ions were chosen as potential interfering species during the analytical process by using DPASV under the above optimized experimental parameters. The interference experiments were performed by detecting 2.0 μM Cu^2+^, Cd^2+^, and Hg^2+^ in 0.1 M PBS (pH 6.5) containing 10 μM of the interference ions including K^+^, Ca^2+^, Zn^2+^, Fe^3+^ and Co^2+^ and the changes of DPASV response current signals of the three heavy metal ions after adding the five interference ions are shown in Fig. 7A. The results showed that the peak currents of Cu^2+^, Cd^2+^, and Hg^2+^ decreased slightly after the addition of interfering metal ions, which may be due to the binding ability of these interfering metal ions to PGA. However, as seen from the Fig. 7A, the decrease of the peak currents of the three heavy metal ions is less than 20% in the presence of the interference ion with 5-fold the detected ion concentration, indicating that PG/GCE exhibits high selectivity for Cu^2+^, Cd^2+^, and Hg^2+^ in the DPASV analysis. Fig. 7B shows the changes of DPASV response current signals of 2.0 μM Cu^2+^, Cd^2+^, and Hg^2+^ in 0.1 M PBS (pH 6.5) after adding 10 μM K^+^, Ca^2+^, Zn^2+^, Fe^3+^ and Co^2+^. The decrease of the peak currents of the three heavy metal ions is less than 25%, indicating that PG/GCE exhibits high selectivity for Cu^2+^, Cd^2+^, and Hg^2+^.

### 3.6. Stability and reproducibility of PG/GCE

**Fig. 8:**
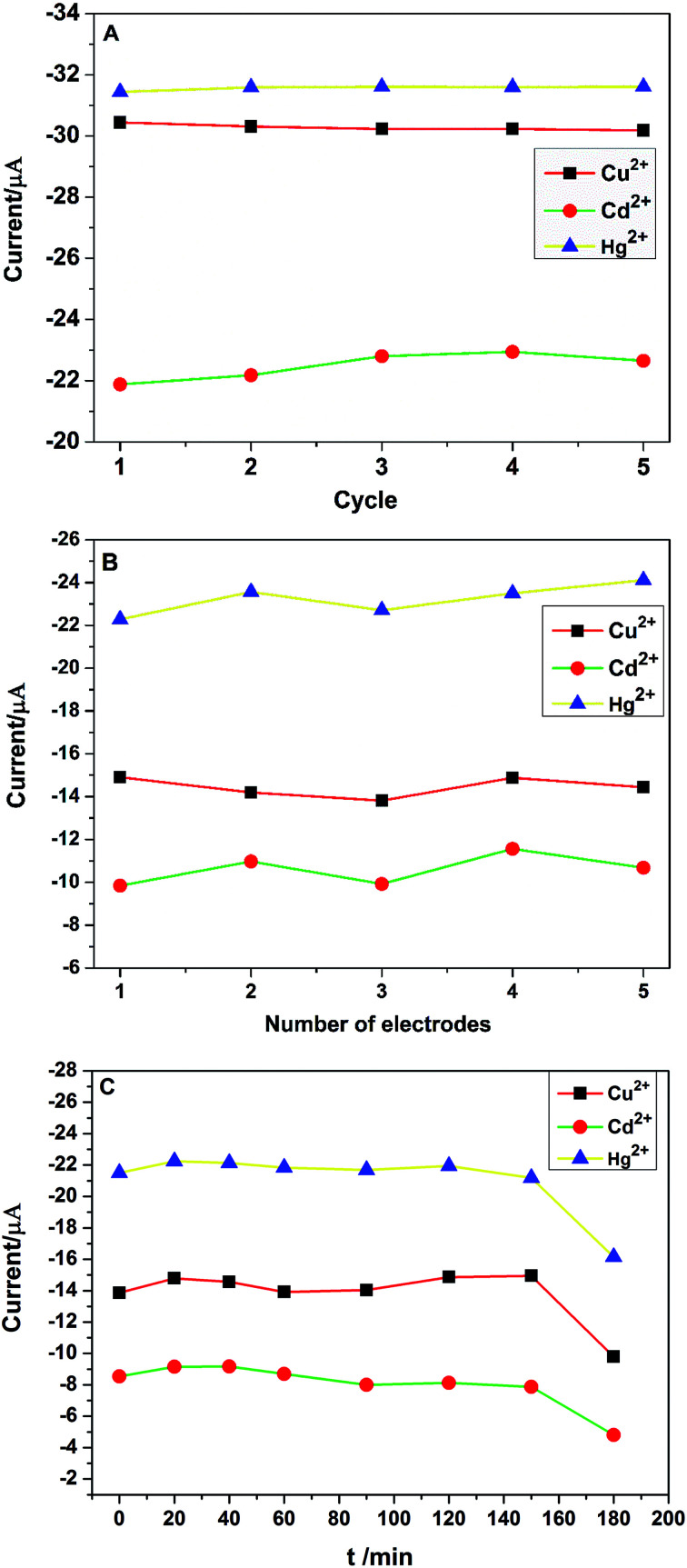
(A) The oxidation peak current changes of PG/GCE for 5 repetitive measurements in 0.1 M PBS (pH 6.5) containing 2.0 μM Cu^2+^, Cd^2+^ and Hg^2+^. Scan rate: 0.1 V s^−1^; (B) the changes of oxidation peak current in 0.1 M PBS (pH 6.5) containing 2.0 μM Cu^2+^, Cd^2+^ and Hg^2+^ with five different electrodes prepared in parallel. (C) The changes of oxidation peak current in 0.1 M PBS (pH 6.5) containing 2.0 μM Cu^2+^, Cd^2+^and Hg^2+^ with different soaking time of PG/GCE before initiating the measurement.

Stability and reproducibility are two important performance properties of electrochemical sensors. The stability of PG/GCE was examined by 5 repetitive measurements of 2.0 μM Cu^2+^, Cd^2+^, and Hg^2+^ in 0.1 M PBS using CV at the same PG/GCE electrode under the optimized experimental conditions. The change of redox peak currents is not obvious and the oxidation peak currents of the three target heavy metal ions are shown in Fig. 8A, with relative standard deviation (RSD) values of 0.34% for Cu^2+^, 1.98% for Cd^2+^ and 0.23% for Hg^2+^, respectively. Furthermore, the PG/GCE electrode was stored in the refrigerator at 5 °C for two weeks, and the oxidation peak currents for Cu^2+^, Cd^2+^ and Hg^2+^ remained at 96.8%, 95.1% and 97.3% of their initial values, respectively. The obtained results indicated the good stability of the modified electrode PG/GCE. The reproducibility of the modified electrodes was also checked with five different PG/GCE electrodes prepared using the same conditions. Fig. 8B depicts the oxidation peak current changes of five different electrodes in simultaneous detection of 2.0 μM Cu^2+^, Cd^2+^, and Hg^2+^ in 0.1 M PBS under the optimized conditions. The results show that the modified electrode also has good reproducibility with the RSD of the peak currents as 3.23% for Cu^2+^, 6.85% for Cd^2+^, 3.14% for Hg^2+^, respectively. Under the optimized conditions, the changes of the oxidation peak currents with increasing the soaking time of PG/GCE in 0.1 M PBS containing 2.0 μM Cu^2+^, Cd^2+^, and Hg^2+^ before initiating measurements are shown in Fig. 8C. When the soaking time less than 150 minutes, the PG/GCE has good stability with the RSD of the peak currents as 3.28% for Cu^2+^, 5.86% for Cd^2+^, 1.69% for Hg^2+^, respectively.

### 3.7. Practical application of PG/GCE

In order to evaluate the high sensitivity and practical value of the modified electrode, PG/GCE was used to determine Cu^2+^, Cd^2+^, and Hg^2+^ concentration in lake water samples. Lake samples were pretreated by filtration and then added to 0.1 M PBS, finally detected using the DPASV method under the optimized conditions. Because of the low concentrations of Cu^2+^, Cd^2+^, and Hg^2+^ in the lake samples, standard addition method was used to analyze Cu^2+^, Cd^2+^, and Hg^2+^ concentration in the lake samples. The recovery results for Cu^2+^, Cd^2+^, and Hg^2+^ are summarized in Table 2. The recoveries of the three heavy metal ions are almost 100% (99.3–100.4%), demonstrating that the prepared PG/GCE can be used to analyze the actual samples with high sensitivity and accuracy. Additionally, ICP-MS was also used to verify the accuracy of PG/GCE on the analysis results of real samples. As summarized in Table 3, the results show that the Cu^2+^, Cd^2+^, and Hg^2+^ concentration obtained by two methods are consistent, and the relative error was below 3.1%. It is proved that PG/GCE has the application prospect of accurately detecting heavy metal ions in the environment.

**Table 2:** Detection results of Cu^2+^, Cd^2+^, and Hg^2+^ in lake water sample at PG/GCE

Analytes	Added (μM)	Found (μM)	Recovery (%)
Cu^2+^	0	—	—
	0.3	0.299	99.7
	0.5	0.501	100.2
Cd^2+^	0	—	—
	0.3	0.298	99.3
	0.5	0.502	100.4
Hg^2+^	0	—	—
	0.3	0.301	100.3
	0.5	0.449	99.8

**Table 3:** Detection results of Cu^2+^, Cd^2+^, and Hg^2+^ concentration in lake water sample

Analytes	Added (ppb)	By this method (ppb)	By ICP-MS (ppb)	Relative error
Cu^2+^	8.0	7.872	8.018	−1.8%
Cd^2+^	8.0	8.176	8.012	2.1%
Hg^2+^	8.0	7.813	8.064	−3.1%

## 4. Conclusion

In summary, the glassy carbon electrode (GCE) modified by PGA/GO composite film (PG) displayed high stability and good reproducibility for the simultaneous detection of heavy metal ions Cu^2+^, Cd^2+^, and Hg^2+^ by using DPASV method. The peak potentials of each heavy metal were well defined and sufficiently separated during the simultaneous determination of the three heavy metal ions. Due to the high affinity of PGA to heavy metal ions and the good conductivity of GO, the prepared PG/GCE electrode exhibited very high electrochemical response for the detection of the three heavy metal ions, with high sensitivity of superior to most of the reported values. Moreover, lake water detection also confirmed that the prepared PG/GCE electrode was suitable for the detection of heavy metal ions in practical samples, demonstrating the modified electrode could be used as a promising platform for the monitor and detection of heavy metal ions in environmental applications.

## Author contributions

The manuscript was written through contributions of all authors. All authors have given approval to the final version of the manuscript. Wei Yi and Xiaohua He designed research; Wei Yi performed research and analyzed data; Zihua He prepared poly(γ-benzyl-glutamate); Junjie Fei gave some suggestions; and Wei Yi and Xiaohua He wrote the paper.

## Conflicts of interest

There are no conflicts to declare.

## Supplementary Material

RA-009-C9RA01891C-s001
