# Supplementary material for: Sensitive electrochemical sensor based on poly(l-glutamic acid)/graphene oxide composite material for simultaneous detection of heavy metal ions
Source: RSC Adv. 2019 Jun 3;9(30):17325–34. doi: 10.1039/c9ra01891c (PMC9064582; doi:10.1039/c9ra01891c)
Supplement: RA-009-C9RA01891C-s001 [file RA-009-C9RA01891C-s001.pdf]

Electronic Supplementary Material (ESI) for RSC Advances.
This journal is © The Royal Society of Chemistry 2019

Supporting Material

Sensitive Electrochemical Sensor Based on Poly(L-glutamic acid)/Graphene Oxide Composite Material for Simultaneous Detection of Heavy Metal Ions

Wei Yi, [a]Zihua He, [a] JunjieFei, [b] Xiaohua He* [a]

[a] School of Chemistry and Molecular Engineering, East China Normal University, 500 Dongchuan Road, Shanghai 200241, China

Email: xhhe@chem.ecnu.edu.cn

[b] Key Laboratory of Environmentally Friendly Chemistry and Applications of Ministry of Education, College of Chemistry, Xiangtan University, Xiangtan 411105, China

Experimental Section

Reagents and Materials

(4-aminophenyl)-10,15,20-tris(phenyl)porphyrin(99.0%) and γ-Benzyl-L-glutamate (99.0%) was purchased from Sigma-Aldrich. Trifluoroacetic acid (TFA, A.R. grade), N,N-dimethylformamide (DMF, A.R. grade),and other solvents were purchased from Shanghai Chemical Reagent and purified by conventional procedures if needed.γ-Benzyl-L-glutamate N-carboxyanhydride (BLG-NCA) was synthesized according to the literature[1].

Synthesis of Polypeptide

The synthetic route of poly (L-glutamic acid) (PGA) is as follows:

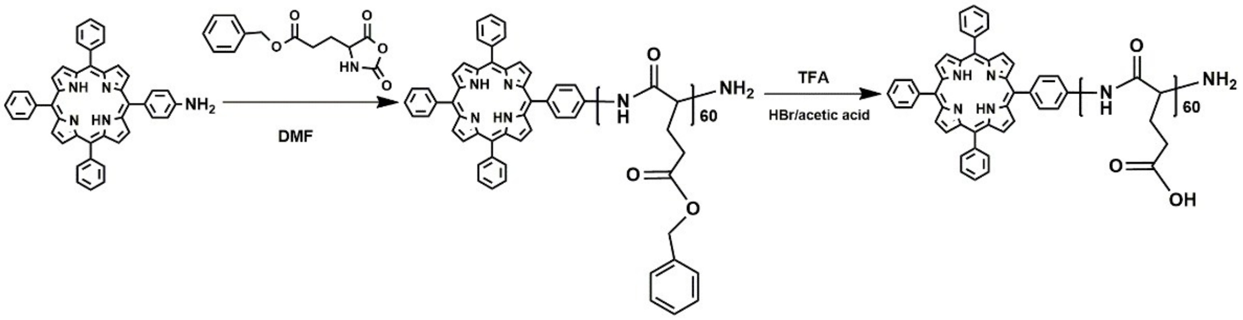

1. Synthesis of Poly (γ-benzyl-glutamate)

Poly (γ-benzyl-glutamate) (PBLG) with a terminal porphyrin group was synthesized by using 5-(4-aminophenyl)-10,15,20-tris(phenyl)porphyrin as an initiator and the

synthetic procedure was adapted from the literature[2].In a typical experiment, BLG-NCA(3.50g, 13.5mmol), 5-(4-aminophenyl)-10,15,20-tris(phenyl)porphyrin (0.17g, 0.27mmol)and anhydrous DMF (40 mL) were added into a dried round-bottom flask with a magnetic bar in a glove box, and then the solution was stirred under pure nitrogen at room temperature for 5 days. After polymerization, the solution was concentrated and then precipitated into an excess amount of methanol, filtered, and dried at room temperature in a vacuum oven overnight.

Yield: 87.2%, Mn (GPC) = 2.25×10^4, Mw/Mn =1.26. ^{1}H NMR(500 MHz, $CDCl_3$+15%TFA), δ(*ppm*): 8.80~8.00 (m, porphyrin), 7.88(br, N***H***CO), 7.31 (br, $C_6\boldsymbol{H}_5CH_2$–), 5.07 (br, $C_6H_5C\boldsymbol{H}_2$–), 4.61(br, –OCC***H***NH–), 2.50~2.42 (m, –OOC$C\boldsymbol{H}_2CH_2$–), 2.13~1.91 (m, –OOC$CH_2C\boldsymbol{H}_2$–). (See Fig.1S and Fig. 2S)

2. Synthesis of poly (L-glutamic acid)

Poly (L-glutamic acid) (PGA) was synthesized according to a published procedure[3].In a typical experiment, the above prepared PBLG (1.0g) was dissolved in TFA (1.2 mL) and 33 wt% HBr/acetic acid (4.5 mL), and then stirred for 2 h at room temperature. The polymer was precipitated by using excess anhydrous diethyl ether, filtered, purified three times from acetic acid to anhydrous diethyl ether, and dried at 40°Cunder vacuum for 48 hours.^{1}H NMR (500 MHz, DMSO-d_6), δ (*ppm*): 12.10 (br, –COO***H***), 7.95 (s,–CHN***H***CO–), 2.89-2.73 (m, –$C\boldsymbol{H}_2C\mathit{H}_2$COOH), 2.09 (s, 1H, –C***H***NHCO–).

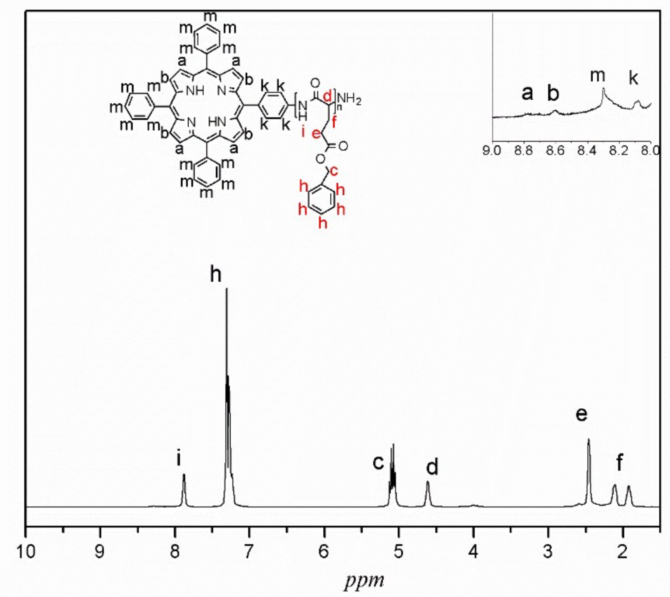

Fig.S1.^{1}H NMR spectrum of poly (γ-benzyl-glutamate).

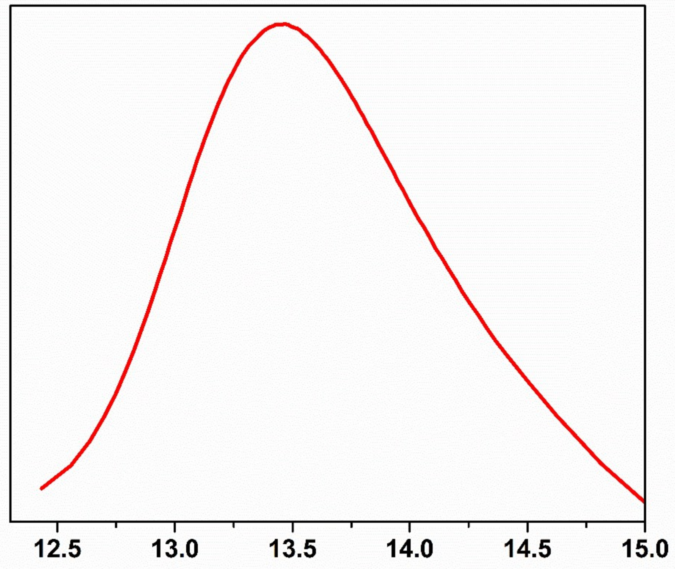

Fig.S2. GPC of poly (γ-benzyl-glutamate).

Reference

[1] E.R. Blout, R.H. Karlson, *J. Am. Chem. Soc.*, **1956**, *78*, 941-946.
[2] X. He, C. Gao, W. Sun, W. Huang, S. Lin, D. Yan, *J. Polym. Sci. Part A: Polym. Chem.,* **2013**,*51*, 1040-1050.
[3] J. Babin, D. Taton, M. Brinkmann, S. Lecommandoux, *Macromolecules*, **2008**,*41,*1384-1392.
